# Plastic ingestion by the Wels catfish (*Silurus glanis* L.): detailed chemical analysis and degradation state evaluation

**DOI:** 10.1016/j.toxrep.2021.11.006

**Published:** 2021-11-11

**Authors:** Matej Mičušík, Angela Kleinová, Mikuláš Oros, Peter Šimon, Tibor Dubaj, Michal Procházka, Mária Omastová

**Affiliations:** a Polymer Institute, Slovak Academy of Sciences, 845 41 Bratislava, Slovakia; b Institute of Parasitology, Slovak Academy of Sciences, Hlinkova 3, 04001 Košice, Slovakia; c Department of Physical Chemistry, Slovak Technical University, Radlinského 9, 812 37 Bratislava, Slovakia

**Keywords:** Plastic waste, plastic ingestion, low-density polyethylene, chemical analysis, degradation

## Abstract

Plastic ingestion by various organisms within different trophic levels, including humans, is becoming a serious problem worldwide. Plastic waste samples are often found concentrated in an organism’s digestive tract and can be degraded and further translocate to the surrounding tissue or circulatory systems and accumulate in food chains. In the present work, we report a detailed chemical analysis and degradation state evaluation of a relatively large piece of plastic waste found in the gastrointestinal tract of a Wels catfish (*Silurus glanis* L.) caught in the Bodrog River (Danube River basin), eastern Slovakia. Chemical analysis by surface-sensitive X-ray photoelectron spectroscopy (XPS) was performed to identify the surface composition of the digested plastic piece. Micro-Fourier transform infrared (μFTIR) spectroscopy showed that the plastic waste was oxidized low-density polyethylene (LDPE), with some nylon fibers adhered on the surface. Glyceraldehyde adhered onto LDPE was also detected, which might come from the carbohydrate metabolism of that fish. A morphology study by digital optical microscopy indicated solid inorganic particles attached to the surface of LDPE. A degradation study by differential scanning calorimetry (DSC) showed considerable oxidation of LDPE, leading to fragmentation and disintegration of the plastic waste material.

## Highlights

• Large piece of oxidized LDPE was found in the gastrointestinal tract of a catfish.
• Inorganic particles and biotic material were adhered on the surface of plastics.
• DSC showed considerable degradation state of LDPE.

## Graphical abstract

**Figure:**
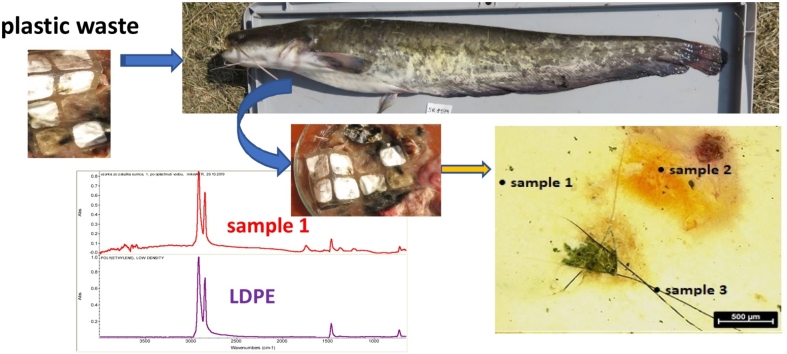

## 1 Introduction

Plastic waste and plastic particles together with other inorganic (Chernyshev et al., 2019; Pikula et al., 2020) or organic particles (Tretyakova et al., 2021) are becoming a serious problem for the environment today. The annual global world plastic production in 2018 reached approximately 359 million tonnes; in Europe, amount was approximately 62 million tonnes (Plastics-the Facts 2019). Approximately 55 % of the world production of plastics encompassed polyolefins, i.e., polypropylene (PP) and polyethylene (PE), followed by polyvinylchloride (PVC) at 16 %, polystyrene (PS) at 7 %, polyethyleneterephthalate (PET) at 7 %, polyurethanes (PUs) at 6 %, with the rest represented by other types of polymers, including polycarbonates (PCs), acrylonitrile butadiene styrene (ABS), polyamides (PAs), etc. Almost 40 % of the plastic production is used for packaging, which means that approximately 140 million tonnes were of very short use (less than one year), producing a massive amount of plastic waste. Plastic waste composition found in nature mimics the composition of plastic industry production, and the most abundant are polyolefins followed by PET, PS and PU, but in nature, one can find all types of plastics as waste (Ragaert et al., 2017). Geyer et al. (Geyer et al., 2017) showed that by 2015, humans had generated 8.3 billion tonnes of plastics and that 6.3 billion tonnes had already become waste. Of this amount, only 9 % was recycled, 12 % was incinerated and 79 % accumulated in landfills or in nature. From these numbers, it is obvious that plastic waste is becoming part of the natural environment, and there is a growing concern regarding pollution brought about by plastic particles (Azevedo-Santos et al., 2019). Currently, various polymers are found in all types of ecosystems, including freshwater, estuarine and marine ecosystems (Law et al., 2010; Lebreton et al., 2017; Sadri and Thompson, 2014). Plastic wastes are observed even in pristine regions as well as in the most isolated islands in the Pacific Ocean (Lavers and Bond, 2017). Ocean contamination is currently a serious problem. The plastic waste entering the ocean comes from the land mostly by river streams (Eerkes-Medrano et al., 2015; Schmidt et al., 2017; Zhang et al., 2015). Plastic items degrade and can fragment into smaller particles (microplastics < 5 mm and mesoplastics 5-25 mm) that remain for long periods in the environment (Andrady, 2017; Fossi et al., 2017). Lechner et al. (Lechner et al., 2014) presented results from a two-year (2010 and 2012) survey on plastic waste transport in the Danube River and showed by using stationary driftnets that the mean plastic abundance and mass is higher than those of drifting larval fish. The plastic input via the Danube into the Black Sea was estimated to be 4.2 t per day.

From the above facts, it is clear that plastics become part of nature and that environmental contamination is a global challenge to ecosystem and human health. Ingestion of plastic waste by animals can cause blockage or internal injuries of the gastrointestinal tract, leading to starvation (Courtene-Jones et al., 2017; Possatto et al., 2011). Recently, numerous studies have documented plastic debris ingestion by mammals (Ribeiro et al., 2019), invertebrates and birds (Lourenço et al., 2017), and both filter feeders and predatory fishes (Santillo et al., 2017). In addition to interfering with and blocking the gastrointestinal tract, plastic materials can release some organic volatile molecules presenting toxicological risks via food chain transfer and bioaccumulation, which can also be dangerous to humans consuming fishes (Chee et al., 1996; Farrell and Nelson, 2013; Hermabessiere et al., 2017; Wright et al., 2013). Moreover, when microplastics exist in water ecosystems, they may accumulate pollutants present such as polychlorinated biphenyls (PCBs) and pesticides that preferentially stick to plastic surfaces (Webb et al., 2012; Skláršová et al., 2007; Simko et al., 2004; Simko et al., 1999). A study by Andrade et al. (Andrade et al., 2019) of the Xingu River Basin in the Amazon revealed the consumption of plastic particles by freshwater fishes in each of the three trophic guilds (herbivores, omnivores, and carnivores). Overall, 80 % of the species analyzed had some plastic particles in their gastrointestinal tracts, ranging from 1 to 15 mm in length. Fourier transform infrared spectroscopy identified 12 kinds of polymers, with 27 % polyethylene, 13 % polyvinyl chloride, 13 % polyamide, 13 % polypropylene, 7 % poly(methyl methacrylate), 7 % rayon, 7 % polyethylene terephthalate, and 13 % of blends from polyamide and polyethylene terephthalate.

The ingestion of plastic by various fishes has been well documented, and articles on the subject have increased considerably over the last decade (Jovanović, 2017); however, there is a lack of information on the rate and extent of plastic degradation processes. In the present work, we focused on the deep analysis of a large piece of plastic waste found in the stomach of a Wels catfish (*Silurus glanis* L.) caught in the Bodrog River (Danube River basin), eastern Slovakia. We performed chemical analysis by Fourier transform infrared (FTIR) spectroscopy and X-ray photoelectron spectroscopy (XPS), morphology studies by digital optical microscopy and degradation state studies by differential scanning calorimetry (DSC). To the best of our knowledge, this is the first study to discuss the degradation state of plastic waste found in the gastrointestinal tract of an animal species with detailed chemical study.

## 2 Materials and Methods

### 2.1 Specimen studied

**Fig. 1:**
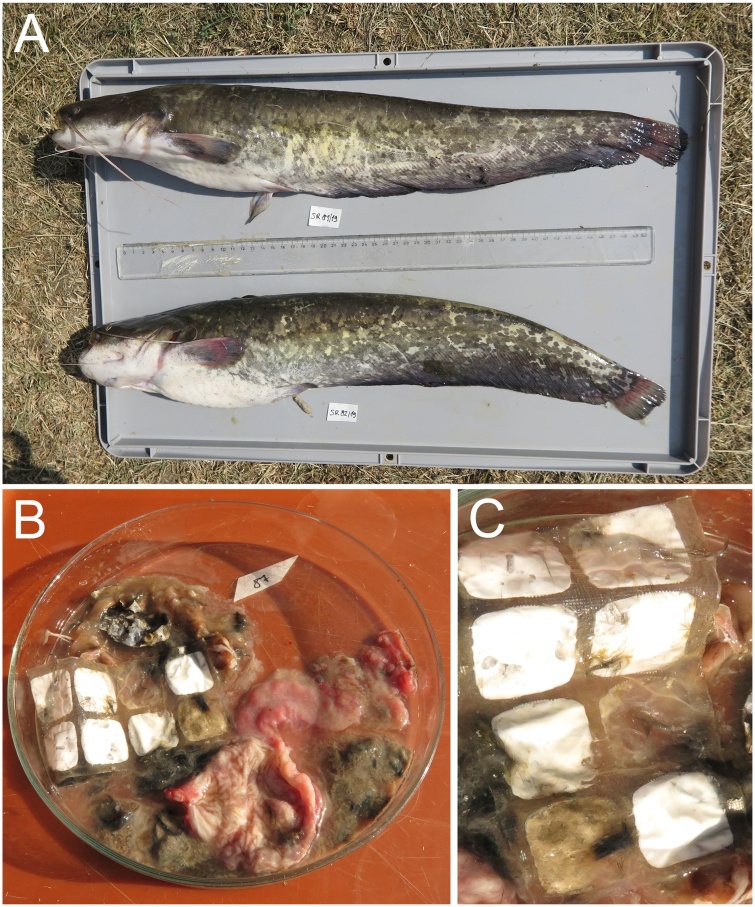
Photograph of a) a Wels catfish (Silurus glanis L.) caught in the Bodrog River, b) piece of plastic waste found in the stomach of the catfish, and c) detail of the relatively large piece of plastic.

Fishing and consumption of fishes within the Bodrog River basin are at one’s own risk due to high concentrations of polychlorinated biphenyls (PCBs) and mercury (Hg), both of which are derived from the Zemplínska Šírava water reservoir (Regulation of the Ministry of Environment of the Slovak Republic). During ichthyoparasitological research, Wels catfish (*Silurus glanis* L.) were examined for parasites by incomplete parasitological necropsy focusing on the organs of the digestive system. Wels catfish are important game fish and food source that are very common representatives of Slovak fish fauna and occur frequently in lowland rivers. A plastic waste sample was found in the stomach of one catfish (total length 57 cm, Fig. 1A) from the Bodrog River near the village Ladmovce (48°24'46.2"N 21°46'59.2"E) in July 2019. Only one specimen was found out of the 17 Wels catfish examined to contain this big piece of plastic. Fish were caught by electrofishing under a permit (No. 62/2019) issued by the Ministry of Environment of the Slovak Republic.

### 2.2 Micro-Fourier Transform Infrared Spectroscopy

Plastic waste samples were analyzed using micro-Fourier transform infrared (μFTIR) spectroscopy with a NICOLET 8700™ spectrophotometer (Thermo Scientific, Madison, USA) using an attenuated total reflection (ATR) accessory. The spectra were measured in the infrared range of 4000–650 cm^−1^ (the measuring window for the used germanium crystal). The FTIR spectra were analyzed using OMNIC™ 8.1 software.

### 2.3 X-ray photoelectron spectroscopy

XPS signals for the studied plastic were recorded using a Thermo Scientific K-Alpha XPS system (Thermo Fisher Scientific, UK) equipped with a microfocused, monochromatic Al Kα X-ray source (1486.68 eV) (more details in Supplementary material).

### 2.4 Digital optical microscopy

Plastic waste samples were analyzed using a Leica DVM6 digital microscope (Leica Microsystems, Germany) using the objective PlanAPO FOV 12.55 with a large magnification range (40 × - 675 ×) at a high working distance (33 mm). For evaluation of the images, LAS X software from Leica was used.

### 2.5 Differential scanning calorimetry

DSC curves were measured using a Perkin Elmer DSC-7 differential scanning calorimeter (USA). The temperature scale of the calorimeter was calibrated to the melting temperatures of In, Sn and Pb, and the calorimetric calibration was based on the melting enthalpy of In. The purge gas forming the reaction atmosphere was oxygen. For both materials, the oxidation induction time (OIT) was measured according to the ISO standard procedure [ISO 11357-6:2018] at 165 °C in oxygen.

## 3 Results and Discussion

The plastic piece found in the stomach of a catfish is depicted in Fig. 1b and c.

Fig. 1. Photograph of a) a Wels catfish (*Silurus glanis* L.) caught in the Bodrog River, b) piece of plastic waste found in the stomach of the catfish, and c) detail of the relatively large piece of plastic.

The plastic waste (7 cm length and 3.5 cm wide, Fig. 1B, and C) obtained from the fish stomach was gently washed and stored at −20 °C until further processing. For the analysis by XPS, we used the sample after washing with water (labeled as Sample 1-aw). In the case of micro-FTIR analysis, different places of the sample were investigated (see discussion below).

For chemical analysis by μFTIR spectroscopy, three different parts were taken from the plastic piece differing in color and structure (Fig. 3b, see discussion to Fig. 3). Sample 1 was a plastic piece covered with contaminants; this sample was also washed with water, and the spectrum was taken before (labeled as Sample 1-bw) and after washing (labeled as Sample 1-aw). Sample 2 represents yellow regions. Sample 3 consisted of black fibers with a thickness of approximately 10 μm.

**Fig. 2:**
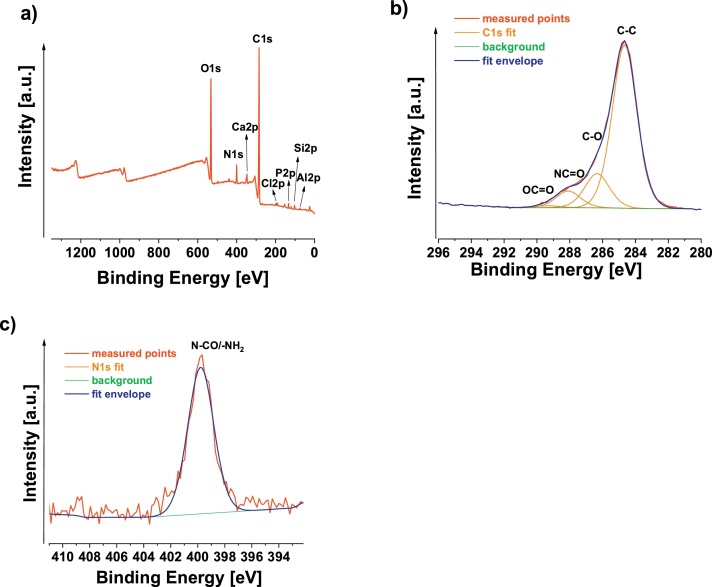
XPS a) survey, b) C1s region, c) N1s region of plastic piece found in the stomach of the catfish.

XPS showed a C1s signal at approximately 284.7 eV, which is typical for organic polymers (Fig. 2, Table 1). It is difficult to identify a specific polymer, but the C1s signal at ∼ 288.0 eV together with the N1s signal at ∼ 399.8 eV indicates the presence of amide (N-CO) or amine (-NH_2_) groups (Beamson and Briggs, 1992). This amide group from the C1s signal (5.4 at.%, Fig. 2b, Table 1) correlates well with the N1s signal corresponding to N-CO/-NH_2_ (4.8 at.%, Fig. 2c, Table 1). These results are a strong indication of some surface layer of peptides and proteins (peptide bond is amidic, N-C = O), which could come from the gastrointestinal tract and are adhered onto the surface of the plastic piece. For phosphorus as PO_4_^3-^ (P2p signal at ∼ 133 eV) and chlorine as chloride (Cl2p signal at ∼ 198.0 eV), it is possible to deduce that they also come from the gastrointestinal environment.

**Fig. 3:**
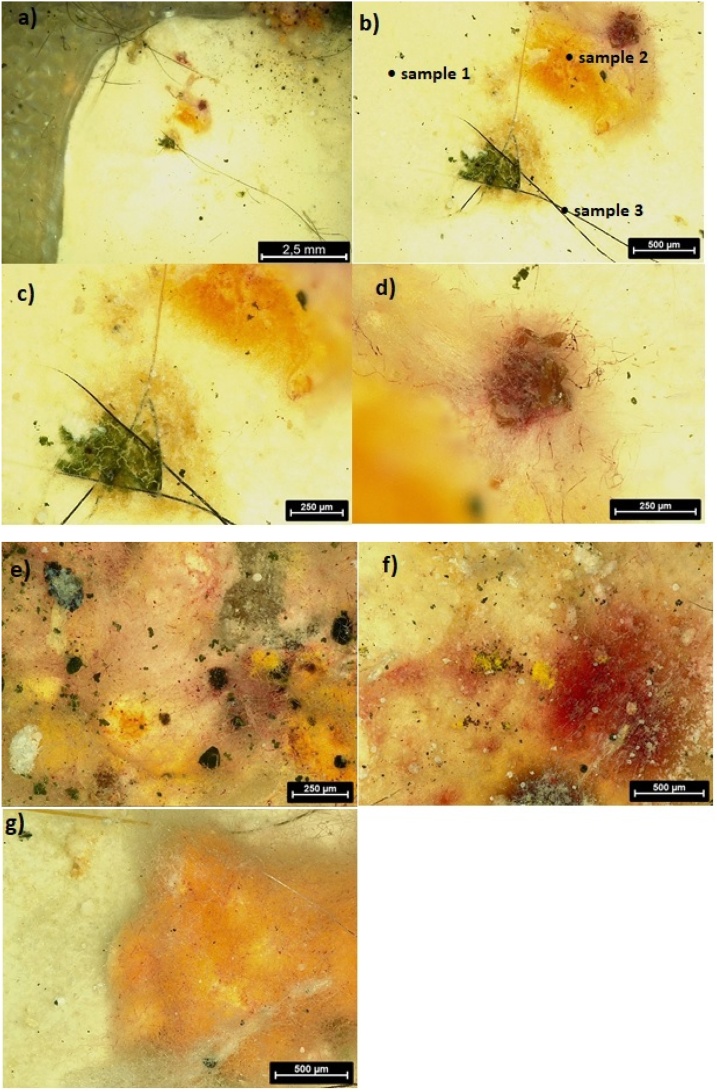
Optical microscopy of a plastic piece in the stomach of the catfish, b) depicts points for IR analysis.

**Table 1:** Apparent surface chemical composition as determined by XPS.

Sample	Chemical surface composition [at. %]								
	C1s C-C/CO/NCO/OCO	O1s	N1s	Si2p	P2p	Al2p	Na1s	Ca2p	Cl2p
**Sample 1-aw**	69.9 52.8/11.1/5.4/0.6	19.8	4.8	1.8	1.4	1.1	0.1	0.9	0.5

Additionally, several other elements on the surface were detected, namely, Si2p (∼102.4 eV), Al2p (∼ 74.2 eV), P2p (∼ 133.0 eV), Ca2p (∼ 347.1 eV), and Cl2p (∼ 197.8 eV). Silicon and calcium are in an oxidized state, probably as some minerals (“Avantage, 2021, version 5.9918; XPS Software Database; Thermo Fisher Scientific Inc., East Grinstead, UK,” n.d.). Aluminum is trivalent Al^3+^. Aluminum is the third most abundant element in Earth’s crust (after O and Si), and it is found in the soil as trivalent Al in the form of minerals such as aluminosilicates or Al oxides (Bojórquez-Quintal et al., 2017). These inorganic minerals most likely come from the soil present in the water and seem to be strongly adhered onto the polymer surface (Fig. 2e).

Black particles (Fig. 3a and e) are most likely inorganic particles coming from the soil present in water. These particles could be some minerals as shown by XPS and discussed above. In addition to these particles, we can see some black fibers, which were further analyzed by μFTIR spectroscopy. Additionally, some yellow and red regions (Fig. 3b, d, f, g) were observed. The red region might be some adhered blood cells or some tissue as a consequence of being in the gastrointestinal tract, but we were not able to study this more precisely with μFTIR spectroscopy because optical microscopy coupled with μFTIR spectroscopy does not precisely show this red region, and we focused only on the yellow regions.

**Fig. 4:**
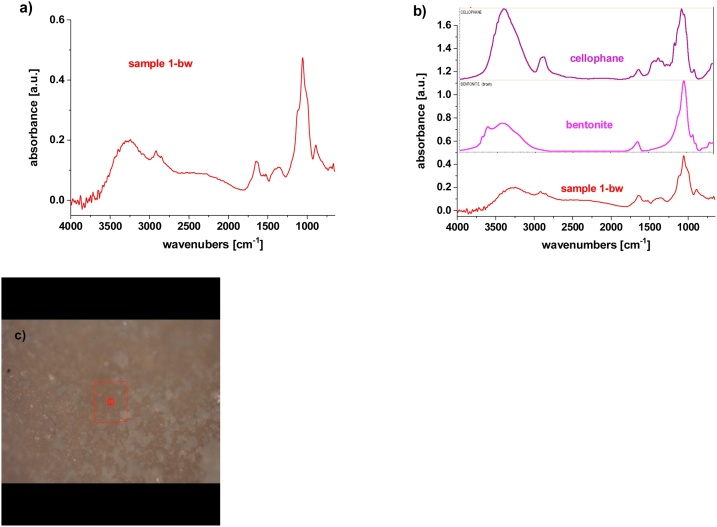
μFTIR spectroscopy of Sample 1 before washing; a) Sample 1-bw, b) comparison with database, and c) micrograph from the location of analysis.

Sample 1-bw (Fig. 4) exhibited the presence of some inorganic material, mostly silicate functionalities, which was also shown by XPS, where the presence of Si-O (silicates, vibrations at 1060 cm^-1^), Ca-O (oxides/minerals, vibrations at 1470-1300 cm^-1^) and Al-O (alumina, vibrations from 3700-3000 and 1275-740 cm^-1^) were detected (Frederickson, 1954).

**Fig. 5:**
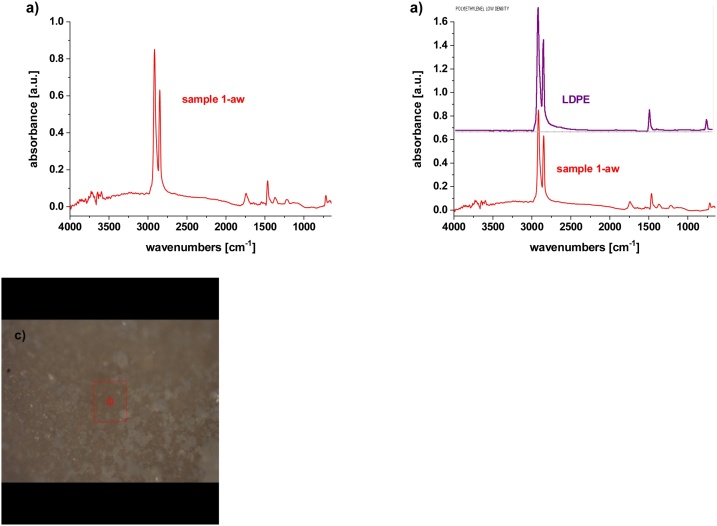
μFTIR spectroscopy of Sample 1 after washing; a) Sample 1-aw, b) comparison with database, c) micrograph from the point of analysis.

After washing, Sample 1-aw was clearly identified as low-density polyethylene (LDPE) (Fig. 5). A strong peak at ∼ 1715 cm^-1^ corresponding to carbonyl groups indicates advanced oxidation and degradation (Jung et al., 2018; Stuart, 2005). The match percentage with reference database of LDPE is in this case ca 98%. This piece of plastic was most likely some packaging made from LDPE, but unfortunately, we were not able to identify the original product to conduct better comparison with the original reference. However, we were able to compare the sample (see the DSC study below) with the degradation behavior of known polyethylene packaging.

**Fig. 6:**
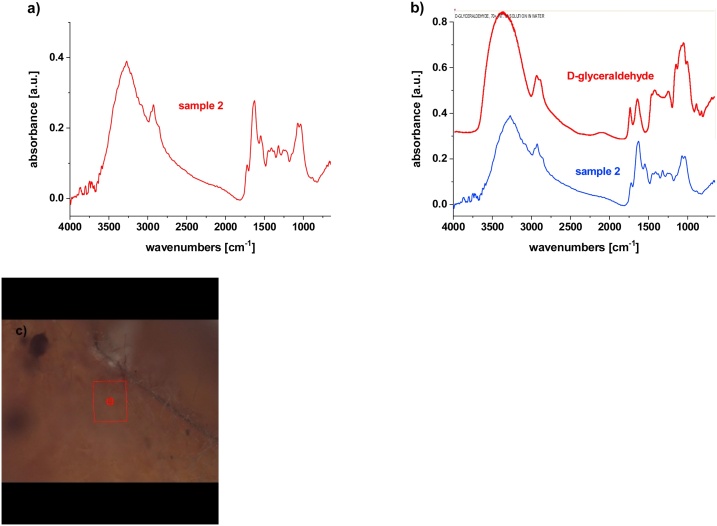
μFTIR spectroscopy of Sample 2; a) Sample 2, b) comparison with database, c) micrograph from the point of analysis.

Sample 2, the yellow part (Fig. 6, Fig. 3), showed good correlation with glyceraldehyde (the match percentage of ca 52%), which might be present due to carbohydrate metabolism in the gut of the catfish. In the spectrum, some protein functional groups were identified as well, which is again a consequence of the plastics being in the gastrointestinal tract. This result is in accordance with the XPS findings discussed above. From the optical microscopy results shown in Fig. 3d, f and g, there is visible yellow and red colored residuum of some organic material as well.

**Fig. 7:**
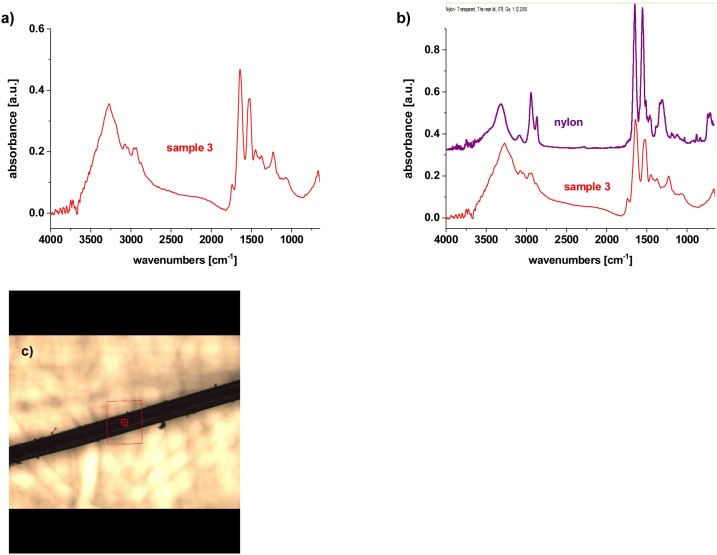
μFTIR spectroscopy of Sample 3; a) Sample 3, b) comparison with database, c) micrograph from the point of analysis.

Sample 3 is a fiber of nylon (polyamide type) (Koenig, 1999), which is confirmed by two typical vibrations at 1642 cm^-1^ (Amide I, C-O_stretch_) and at 1542 cm^-1^ (Amide II, NH_def_). (Fig. 7). The match percentage with reference database of polyamide 6 is in this case ca 60 %. These fibers might come from some textile or fishing yarns; in Fig. 4, they are well visible as black thin fibers.

**Fig. 8:**
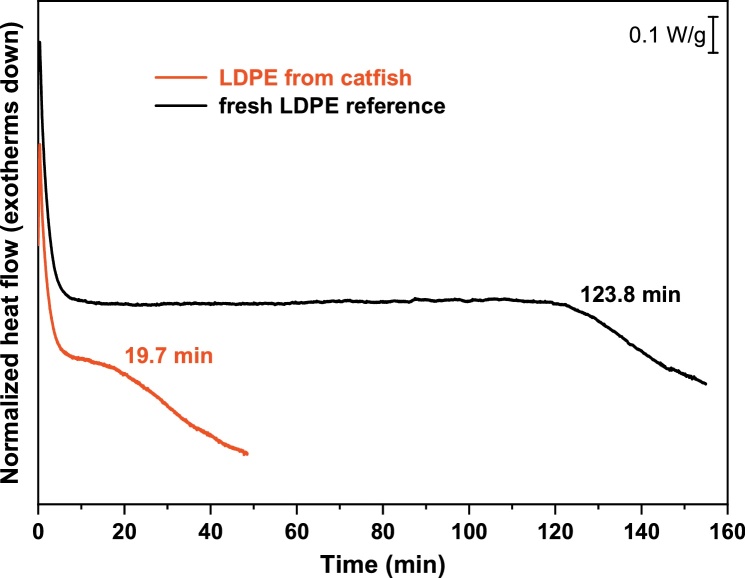
DSC of plastic piece in the gut of the catfish.

Fig. 8 depicts DSC records of LDPE found in the gastrointestinal tract of the catfish compared with a fresh LDPE reference obtained from packaging foil. This reference LDPE has similar stability as was published for nondegraded polyethylene (Šimon and Kolman, 2001). The OIT of the sample taken from the catfish was 19.7 min; the stability of the LDPE packaging foil was 123.8 min. Based on these OIT values, the residual stability, *R*, can be estimated as 19.7/123.8 ≈ 16 % (Šimon and Kolman, 2001). This result suggests that the piece of LDPE found in the catfish was extensively damaged so that it retains less than one-fifth of its initial stability. It should be noted that the DSC results are nonspecific in the sense that we are unable to evaluate the degradation contribution of each individual environmental factor such as UV light exposure before fish ingestion, microbial degradation in water and the final contribution of the environment of the digestive tract after ingestion. However, this extremely low stability means that it can be expected that the material is mechanically unstable, and after a short time, this piece of plastic could be fragmented even after gentle touching. Due to the unsuitable shape of the specimen, it is not possible to evaluate the mechanical properties by standard techniques such as tensile tests or dynamic mechanical analysis.

Chemical analysis indicates the hydrophilization of the original hydrophobic LDPE, which is obvious from the presence of various minerals on the surface detected by XPS and an increase in the number of carbonyl groups as a consequence of oxidation of LDPE chains measured by μFTIR analysis. Additionally, the presence of hydrophilic proteins and glyceraldehydes from carbohydrate metabolism adhered onto the oxidized LDPE (supported by XPS, μFTIR spectroscopy, and optical microscopy) confirms the hydrophilization of the LDPE surface. Generally, the more hydrophilic surfaces, the more are prone to microorganism colonization and thus are more susceptible to the oxidation process due to biotic factors such as enzymes (Kumar Sen and Raut, 2015).

It is very difficult or impossible to define how long that massive fragment was in the digestive tract of the catfish and also to assess the main factor of the degradation. Generally, LDPE is relatively inert and C-C single bonds of LDPE do not undergo hydrolysis and resist photo-oxidative degradation due to the lack of UV-visible chromophores. Adventitious impurities or structural defects present in LDPE as a consequence of final product manufacturing and product of subsequent weathering can act as chromophores. LDPE may contain also various amount of unsaturated C = C bonds (vinylidenes) in the chain, which can be oxidized to hydroperoxides and converted to UV-absorbing carbonyls (Craig et al., 2005). This together with various additives and fillers added to the final polymer are the reasons for very difficult comparison between various LDPE products. Additionally, the rate of degradation depends strongly on the amorphous fraction of the polymer, which also varies between different LDPE products (Chamas et al., 2020). Furthermore, over 400 microbial species were assumptive identified as capable to degrade plastics (Lear et al., 2021). Partial biodegradation was reported also for polyethylene (Ren et al., 2019). Microbiomes usually work together with abiotic factors such as temperature and sun-light, which can initiate changes in the structural integrity of polymers and improve accessibility to enzymatic attack. However, for the majority of commercial plastics including LDPE, clear evidence for microbial degradation remains poor, with a lot of papers failing to truly confirm microbial degradation of synthetic polymers [Lear, 2021]. In our opinion, LDPE fragment found in the gastrointestinal tract of the catfish was at first degraded by abiotic factors and then microbiomes could have done the rest. However, as we have stated above it is not possible to assess the influence of each individual factor and it will be a challenging task to do that even in a controlled study.

Our study shows that ingested plastic not only blocks the digestive tract but also readily interacts with enzymes and thus could influence metabolic processes. Assumably, this totally degraded LDPE piece is mechanically unstable, which could lead to the development of massive fragmentation and consequently the development of micro- and nanopolymer particles entering living organisms.

## 4 Conclusion

XPS and μFTIR studies showed that the plastic piece found in the stomach of a catfish is a swallowed polyethylene packaging. In addition to some inorganic particles on the surface, the polymer was covered with some adhered proteins and residue from carbohydrate metabolism such as glyceraldehyde. These biotic materials were clearly visible in the optical micrographs and confirmed by chemical analysis by XPS and μFTIR spectroscopy. Since the plastic piece was relatively large, it most likely blocked the gastrointestinal tract for a long time (based on the high degradation state of polyethylene) and could also influence the retention of other smaller objects such as nylon fibers, which together with the interaction with proteins and products from carbohydrate metabolism is not beneficial for the quality of life of that particular fish, with still unknown impacts on other aspects such as overall health, among others. DSC determination of the oxidation induction time showed that LDPE from the catfish gut has much lower stability than a new packaging material made of the same polymer. Additionally, this degraded LDPE piece is mechanically unstable, which could lead to the development of micro- and nanopolymer particles entering living organisms. In the future, it would be of interest to quantify the oxidation processes due to biotic factors such as enzymes present in the gastrointestinal tract of fishes and compare them to abiotic factors such as UV light exposure. It appears that animal ingestion could accelerate the degradation of plastic waste and contribute to faster fragmentation and thus to the deterioration of all aspects connected with micro- and nanoplastic pollution.

## Author statement

MM was the main author and did the analysis by XPS and optical microscopy. AK has performed FTIR and the manuscript writing. MO has done the ichthyoparasitological research and the manuscript writing. PS has performed DSC and discussed the degradation stability and contributed to the manuscript writing. TD has done DSC experiments and contributed to the manuscript writing. MP has performed optical microscopy and contributed to the manuscript writing. MO was the project leader and did the manuscript writing.

## Declaration of Competing Interest

The authors report no declarations of interest.
