# Supplementary material for: Plastic ingestion by the Wels catfish (Silurus glanis L.): detailed chemical analysis and degradation state evaluation
Source: Toxicol Rep. 2021 Nov 11;8:1869–76. doi: 10.1016/j.toxrep.2021.11.006 (PMC8609109; doi:10.1016/j.toxrep.2021.11.006)
Supplement: Supplementary file 1 [file mmc1.docx]

**SUPPLEMENTARY MATERIAL**

**Plastic ingestion by the Wels catfish (*Silurus glanis* L.): detailed chemical analysis and degradation state evaluation**

Matej Mičušík^1*^, Angela Kleinová^1^, Mikuláš Oros^2^, Peter Šimon^3^, Tibor Dubaj^3^,
Michal Procházka^1^, Mária Omastová^1^

*^1^ Polymer Institute, Slovak Academy of Sciences, 845 41 Bratislava, Slovakia*

*^2^ Institute of Parasitology, Slovak Academy of Sciences, Hlinkova 3, 04001 Košice, Slovakia*

*^3^ Department of Physical Chemistry, Slovak Technical University, Radlinského 9, 812 37 Bratislava, Slovakia*

*corresponding author: [matej.micusik@savba.sk](mailto:matej.micusik@savba.sk)

# Materials and Methods

## X-ray photoelectron spectroscopy

XPS signals for the studied plastic waste were recorded using a Thermo Scientific K-Alpha XPS system (Thermo Fisher Scientific, UK) equipped with a microfocused, monochromatic Al Kα X-ray source (1486.68 eV). An X-ray beam of 400 μm size was used at 6 mA × 12 kV. The spectra were acquired in constant analyzer energy mode with a pass energy of 200 eV for the survey. Narrow regions were collected with a pass energy of 50 eV. Charge compensation was achieved with the system flood gun. Thermo Scientific *Avantage* software, version 5.9918 (Thermo Fisher Scientific), was used for digital acquisition and data processing. Spectral calibration was determined by using the automated calibration routine and the internal Au, Ag and Cu standards supplied with the K-Alpha system. The surface composition (in atomic %) was determined by considering the integrated peak areas of detected atoms and the respective sensitivity factors. The fractional concentration of a particular element A was computed using the following formula:

$\% A = \frac{{I_{A}}/{s_{A}}}{\sum({I_{n}}/{s_{n}})} \times100 \%$ (1)


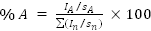

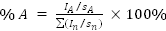


where *In* and *sn* are the integrated peak areas and the Scofield sensitivity factors corrected for the analyzer transmission, respectively.

Figure 1S. Micrograph from an XPS analysis point on the plastic piece found in the stomach of the catfish.

XPS is a surface-sensitive technique that obtains information from the outermost layer of the sample (approximately 10 nm) (Watts and Wolstenholme, 2003); the analyzed area is depicted in Fig. S1. XPS obtains information by detecting the ejected core electrons of a certain atom. From the characteristic peaks, we are able to identify almost all elements, and owing to the chemical shift, it is also possible to identify the chemical state of the atoms. As the method is surface sensitive, it is very prone to surface contamination, so with XPS, it is also possible to see what has been adsorbed on the surface of plastic waste.

## Micro-Fourier Transform Infrared Spectroscopy

µFTIR spectroscopy takes the information from approximately 5 square microns of the sample surface with the approximate 1 µm depth and can be taken as a volumetric technique (statistically relevant for the whole sample). Infrared spectroscopy exploits the fact that molecules absorb frequencies that are characteristic of their structure. Then, according to the position of peaks at certain frequencies, it is possible to identify certain chemical bond structures. Infrared spectroscopy is probably the oldest of the spectroscopic methods used in polymer science (Hummel, 1969; Zerbi, 1999). The FTIR analysis showed that with a proper database (a large number of spectra of known polymers and polymer composites), it is possible to identify most of the unknown plastics.

# References

Hummel, D., 1969. Infrared analysis of polymers, resins, and additives an atlas, [English ed.]. ed. Wiley-Interscience, New York.

Watts, J.F., Wolstenholme, J., 2003. An Introduction to Surface Analysis by XPS and AES, An Introduction to Surface Analysis by XPS and AES. John Wiley & Sons, Ltd, Chichester, UK. https://doi.org/10.1002/0470867930

Zerbi, G. (Ed.), 1999. Modern Polymer Spectroscopy, Modern Polymer Spectroscopy. Wiley-VCH Verlag GmbH, Weinheim, Germany. https://doi.org/10.1002/9783527613922
